# Conversion Surgery after Chemotherapy in a Stage IV *BRAF* V600E‐Mutated Laterally Spreading Tumor With Neuroendocrine Component

**DOI:** 10.1002/deo2.70283

**Published:** 2026-02-04

**Authors:** Reona Kawamura, Naoya Toyoshima, Masau Sekiguchi, Hiroyuki Takamaru, Masayoshi Yamada, Nozomu Kobayashi, Hidekazu Hirano, Yasuyuki Takamizawa, Taiki Hashimoto, Yutaka Saito

**Affiliations:** ^1^ Endoscopy Division National Cancer Center Hospital Tokyo Japan; ^2^ Department of Gastrointestinal Medical Oncology National Cancer Center Hospital Tokyo Japan; ^3^ Colorectal Surgery Division National Cancer Center Hospital Tokyo Japan; ^4^ Diagnostic Pathology Division National Cancer Center Hospital Tokyo Japan

**Keywords:** BRAF, conversion surgery, laterally spreading tumor granular type (LST‐G), neuroendocrine carcinoma (NEC), systemic chemotherapy

## Abstract

We report a rare case of a *BRAF* V600E‐mutated laterally spreading tumor, granular type (LST‐G), with a neuroendocrine carcinoma (NEC) component in a patient with stage IV colorectal cancer. The patient presented with multiple lymph nodes and liver metastases. Following systemic chemotherapy, significant regression of both the primary lesion and metastases was achieved, enabling successful conversion surgery. Postoperative pathological analysis post‐surgery revealed only well‐differentiated tubular adenocarcinoma, with complete disappearance of the NEC component. Molecular testing confirmed the persistence of the *BRAF* V600E mutation. The patient remains recurrence‐free two years after surgery. This case highlights the potential for conversion surgery in stage IV *BRAF*‐mutated colorectal cancer with NEC.

## 1 Introduction

Laterally spreading tumor (LST) is a distinct morphological subtype of colorectal neoplasm characterized by a lateral spreading tumor sized 10 mm or larger. They are classified into LST‐granular type (LST‐G) and LST non‐granular type (LST‐NG) according to the presence or absence of granules [1, 2]. Genetic studies of colorectal cancer have clarified its molecular basis and informed treatment strategies [3]. On the other hand, there are a small number of reported cases of genetic mutation in LSTs. Currently, the known issues are, while LST‐Gs commonly harbor *KRAS* mutations, *BRAF* mutations are rare [4, 5, 6, 7]. We have encountered a unique case of *BRAF* V600E‐mutated LST‐G, which we report in detail together with a review of the literature.

## 2 Case Presentation

**FIGURE 1:**
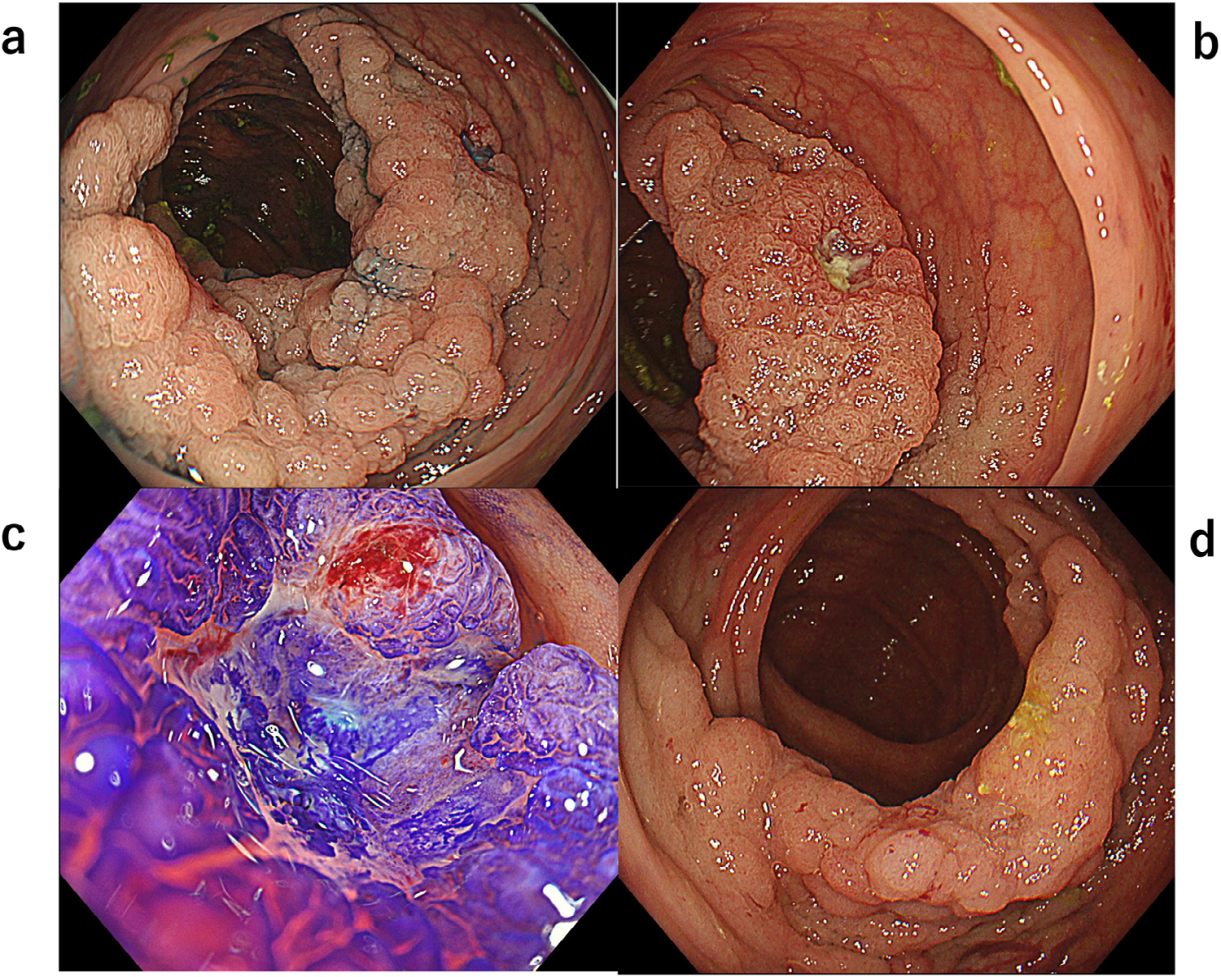
Endoscopic images of the lesion. (a) The lesion was a laterally spreading tumor granular type (LST‐G), and extended over three‐quarters of the circumference. (b) An erosive area is seen. (c) Crystal violet staining showed pit V_N_ at the erosive area. (d) Erosive depression became indistinct after chemotherapy.

**FIGURE 2:**
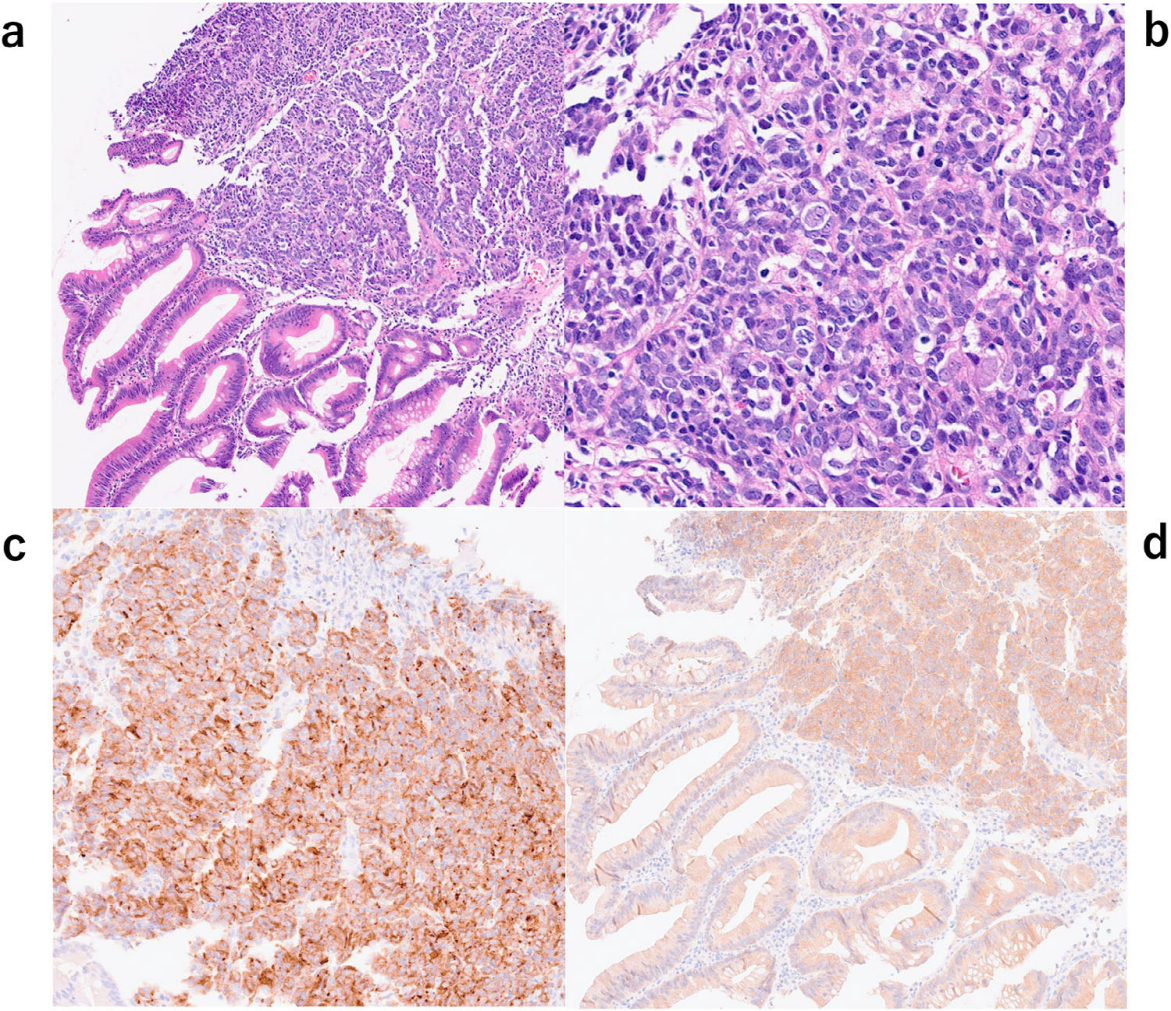
Histopathological images of biopsy specimens from the lesion. (a) Histological image of a biopsy from an erosive area (H‐E stain): neuroendocrine carcinoma (NEC) and well‐differentiated tubular adenocarcinoma components were observed. (b) Histological image of the NEC component (H‐E stain). (c) Diffuse expression of synaptophysin was present in the NEC component. (d) BRAF V600E was positive in both the adenoma and NEC components.

**FIGURE 3:**
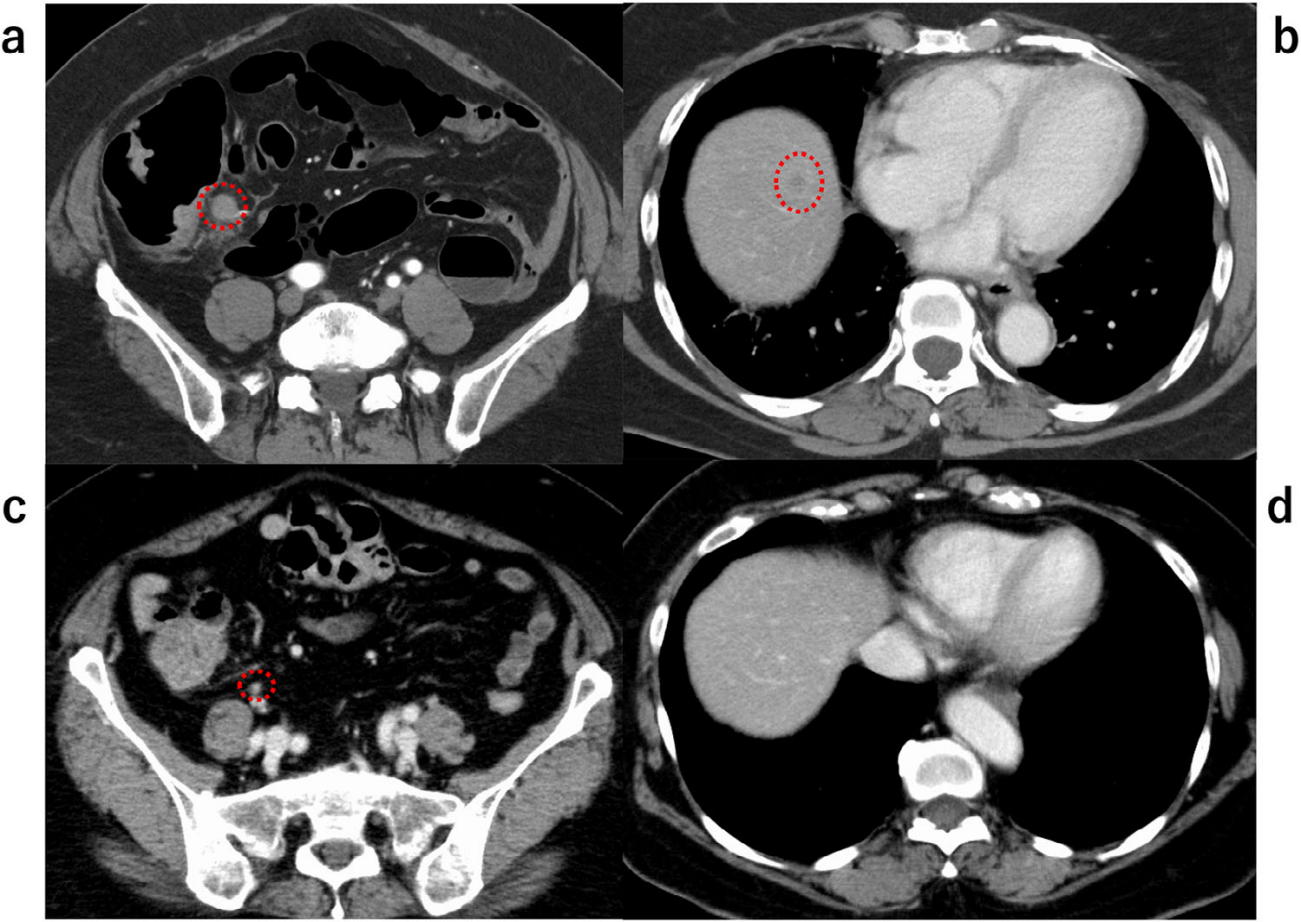
Comparison of lymph node and liver metastasis on computed tomography (CT) scan before and after chemotherapy. (a) Lymph node metastasis before chemotherapy. (b) Liver metastasis before chemotherapy. (c) Lymph node metastasis after chemotherapy, showing regression. (d) Liver metastasis after chemotherapy, showing regression.

A woman in her 60s underwent a colonoscopy after a positive fecal occult blood test, revealing a LST‐G in the ascending colon. She had no significant medical history other than resolved purpura rheumatica and psoriasis. Endoscopic examination identified a 90‐mm 0‐Is+IIa lesion extending over three‐quarters of the colonic circumference, with a small erosive depression demonstrating JNET type 3 and pit pattern Vn features (Figure 1a–c). Biopsies from the depression revealed a neuroendocrine carcinoma (NEC) component coexisting with well‐differentiated tubular adenocarcinoma (Figure 2a–c). BRAF V600E immunostaining was positive for the entire lesion (Figure 2d). Molecular analysis further confirmed a *BRAF* V600E mutation with a microsatellite‐stable phenotype. Staging computed tomography (CT) scans revealed multiple lymph node and liver metastases (cT1bN2M1) (Figure 3a,b). She was initially planned to undergo endoscopic submucosal dissection (ESD), but based on the clinical diagnosis, the treatment plan was switched to systemic chemotherapy with etoposide plus cisplatin (EP regimen). After two cycles of chemotherapy, CT showed regression of the primary tumor and metastases. Subsequent colonoscopy demonstrated no significant change in tumor size and a less distinct erosive depression (Figure 1d). Biopsies revealed only tubular adenocarcinoma, with no residual NEC component. After six total cycles, the disease was downstaged to ycT1bN0M0 (Figure 3c,d).

The patient underwent laparoscopic right hemicolectomy with D2 lymphadenectomy. Pathological examination revealed well to moderately differentiated tubular adenocarcinoma (pTis), no lymphovascular invasion, and negative margins. BRAF V600E immunostaining was positive for the surgical specimen, and molecular testing also revealed the presence of the *BRAF* V600E mutation. No lymph node metastases were identified. The patient remains recurrence‐free at two years without adjuvant therapy.

## 3 Discussion

This is the first report of a *BRAF* V600E‐mutated LST‐G with an NEC component supported by detailed endoscopic and histopathological findings. While LST‐Gs commonly harbor *KRAS* mutations, *BRAF* mutations are rare, reported in only 2.7% (4/149) of cases (Table 1, [4, 5, 6, 7]). This case raises two hypotheses. First, *BRAF* V600E‐mutated advanced colorectal cancers are thought to originate from SSLs [8]. A sessile serrated lesion with dysplasia (SSLD) is defined as an SSL that contains a dysplastic area. Sometimes, the dysplastic components of SSLDs are observed in adenomatous findings, so this case may have originated as an SSLD, subsequently losing its serrated component. Second, an alternative pathway may exist for *BRAF* V600E‐mutated LST‐G.

**TABLE 1:** Summary of reported laterally spreading tumor granular type (LST‐G) cases tested for *KRAS* and *BRAF* mutations.

Study, year	Lesion, *n*	Sex, M/F	Age mean (range)	Size, mean (range)	Location, (L/R)	Pathological Depth (adenoma/Tis,T1)	*BRAF* mutations, *n* (%)	*KRAS* mutations, *n* (%)
Sugimoto [4], 2010	35	18/17	65 ± 13	37 ± 16	15/20	^a^4/31	2 (6%)	19 (54%)
Sakai [5], 2014	51	24/27	67 ± 12	41 ± 20	27/24	28/23	2 (4%)	34 (67%)
Konda [6], 2014	25	12/13	71 (55–89)	29 (12–73)	12/13	9/16	0	17 (68%)
Sugai [7], 2017	38	25/13	(50–83)	19 (11–30)	21/17	8/30	0	27 (71%)
Present study, 2024	1	0/1	61	93	0/1	Unknown (cT1b)	1	0

^a^ One case contained a serrated adenoma.

The dramatic chemotherapy response and disappearance of the NEC component allowed for conversion surgery, an uncommon approach in stage IV colon NECs, which typically have a poor prognosis [9, 10]. The colonoscopy before chemotherapy revealed the NEC component from biopsies of the depressed area. Endoscopic findings outside the depressive area indicated early‐stage colorectal cancer. The NEC is thought to have originated from the adenocarcinoma and to have caused the metastases. Comparing the malignancy of well‐differentiated tubular adenocarcinoma and NEC, chemotherapy targeting the NEC was selected. Colorectal primary NECs are routinely treated with the EP regimen [9, 10]. Despite the poor prognosis with the EP regimen showing a response rate of only 30%–40% [10], this favorable outcome may be attributable to the limited extent of the NEC component, as suggested by the pre‐treatment endoscopic findings. Notably, NECs containing adenomas have been reported to be associated with better prognosis [10]. Some cases were reported in which colon primary NEC led to conversion surgery after successful chemotherapy [10], but there is no clear definition of conversion surgery yet, and we expect that this case report will lead to a promising outcome for determining the indications for conversion surgery. This case highlights the potential for conversion surgery in stage IV *BRAF*‐mutated colorectal cancers with NEC.

## Author Contributions

**Reona Kawamura**: conceptualization, investigation, writing – original draft, and methodology. **Naoya Toyoshima**: supervision and writing – review and editing. **Masau Sekiguchi**: writing – review and editing. **Hiroyuki Takamaru**: writing – review and editing. **Masayoshi Yamada**: writing – review and editing. **Nozomu Kobayashi**: writing – review and editing. **Hidekazu Hirano**: writing – review and editing. **Yasuyuki Takamizawa**: writing – review and editing. **Taiki Hashimoto**: supervision, writing – review and editing, and methodology. **Yutaka Saito**: supervision and conceptualization.

## Conflicts of Interest

The authors declare no conflicts of interest.

## Funding

This research has not received funding from any company.

## Ethics Statement

This study was approved by the Ethics Committee of National Cancer Center Hospital (approval No. [2022‐382]). Verbal informed consent was obtained from the patient, and anonymity has been preserved. The study was conducted in accordance with the Declaration of Helsinki (2013 revision). We preserved individual personal information and made every effort to protect privacy.
